# IL-12-Induced Immune Suppressive Deficit During CD8+ T-Cell Differentiation

**DOI:** 10.3389/fimmu.2020.568630

**Published:** 2020-10-28

**Authors:** Pranav S. Renavikar, Sushmita Sinha, Ashley A. Brate, Nicholas Borcherding, Michael P. Crawford, Scott M. Steward-Tharp, Nitin J. Karandikar

**Affiliations:** Department of Pathology, University of Iowa Health Care, Iowa City, IA, United States

**Keywords:** immune regulation, CD8+ T cells, suppressor cells, autoimmunity, Tregs

## Abstract

Autoimmune diseases are characterized by regulatory deficit in both the CD4+ and CD8+ T-cell compartments. We have shown that CD8+ T-cells associated with acute relapse of multiple sclerosis are significantly deficient in their immune suppressive ability. We hypothesized that distinct CD8+ cytotoxic T-cell (Tc) lineages, determined by cytokine milieu during naïve T-cell differentiation, may harbor differential ability to suppress effector CD4+ T-cells. We differentiated purified human naïve CD8+ T-cells *in vitro* toward Tc0 (media control), Tc1 and Tc17 lineages. Using *in vitro* flow cytometric suppression assays, we observed that Tc0 and Tc17 cells had similar suppressive ability. In contrast, Tc1 cells showed significant loss of suppressive ability against *ex vivo* CD4+ T-cells and *in vitro*-differentiated Th0, Th1 and Th17 cells. Of note, Tc1 cells were also suboptimal in suppressing CD4-induced acute xenogeneic graft versus host disease (xGVHD) *in vivo*. Tc subtypes derived under various cytokine combinations revealed that IL-12-containing conditions resulted in less suppressive cells exhibiting dysregulated cytotoxic degranulation. RNA sequencing transcriptome analyses indicated differential regulation of inflammatory genes and enrichment in GM-CSF-associated pathways. These studies provide insights into the role of T-cell differentiation in CD8 suppressive biology and may reveal therapeutically targetable pathways to reverse suppressive deficit during immune-mediated disease.

## Introduction

As key regulators of the immune response, T-cells can serve both causative and protective roles during immune-mediated damage (1–8). Studies from our group and others have demonstrated an immunoregulatory and disease-suppressive function for CD8+ T-cells in both the autoimmune disease multiple sclerosis (MS) and its animal model (EAE) (9–20). Similar evidence has accumulated in the context of autoimmune diseases such as type 1 diabetes, colitis, SLE-like disease, Graves' disease, among others (21–25). These regulatory CD8+ T-cells function, in part, through suppression of autoreactive CD4+ responses (9, 14, 26–28).

Studies in MS point to a change in immune dynamics during disease relapse periods. We have demonstrated that acute MS relapses are characterized by a substantial deficit in the suppressive ability of patients’ CD8+ T-cells, as well as an increased resistance of patients’ CD4+ T-cells to suppression (29). Intriguingly, these functional deficits normalize during disease remission (30). This suggests that the relapse-associated inflammatory cytokine environment could influence CD8+ T-cells’ suppressive ability.

Similar to CD4+ T-helper (Th) cell subsets, lineages for CD8+ cytotoxic T-cells (Tc), such as Tc0 (media control), Tc1 (IL-2, IL-12, and anti-IL4), Tc17 (IL-6, IL-1β, TGF-β, anti-IL4, and anti-IFNγ), are predominantly determined by the cytokine milieu present during differentiation and defined by certain signature cytokines and transcription factors (31–34). We hypothesized that differentiation of naïve CD8+ T-cells along these pathways would result in variable immune-suppressive potential. Using both *in vitro* assay systems as well as an *in vivo* xenogeneic graft versus host disease (xGVHD) model, we discovered that CD8+ T-cells differentiated toward the Tc1 phenotype had significantly lower suppressive ability. Importantly, this inhibition was associated with IL-12-induced dysregulation of degranulation mechanisms and induction of multiple inflammatory pathways, revealing potential therapeutic targets for the reversal of the suppressive deficit.

## Materials and Methods

### Cell Preparation and Bead Sorting

Peripheral blood mononuclear cells (PBMC) from healthy subjects were isolated from de-identified leukocyte reduction system (LRS) cones containing leukocyte rich whole blood from platelet donors at the University of Iowa, DeGowin Blood Center. PBMC isolation was performed with Ficoll-Paque (GE Healthcare) density gradient centrifugation and frozen in DMSO-containing media for further use. Naïve CD8+ T-cells and/or naïve CD4+ T-cells were isolated from freshly thawed PBMC [RPMI 1640 (Corning, 10-040-CV) with DNase at 10KU/ml (Sigma D4513-1vl)] with manual LS column MACS sorting using human naïve CD8+ T-cell isolation kit (Miltenyi Biotech 130-093-244) and human naïve CD4+ T-cell isolation kit (Miltenyi Biotech 130-094-131) respectively according to manufacturer specifications. Sort purities were routinely above 95% by flow cytometric analysis (**Supplementary Figure 1A**). On the day of suppression assays, autologous CD4+ CD25- cells were sorted from thawed PBMC using human CD4+ T-cell isolation kit (Miltenyi Biotech, 130-096-533) and CD25+ microbeads (Miltenyi Biotech, 130-092-983). T-lymphocyte-depleted PBMC were used as antigen-presenting cells (APC).

### Tc Subset Differentiation

Naïve CD8+ T-cells were sorted from PBMC and resuspended at 1 × 10^6^ cells/ml in Stemline hematopoietic stem cell expansion serum-free media (Sigma, S0192), followed by stimulation in various differentiation conditions (Media Alone/Tc0, Tc1, and Tc17) (31, 35–37) as follows: (1) Media Alone/Tc0: no cytokines/antibodies added; (2) Tc1: anti-IL-4 (7 µg/ml, BD554481), IL-2 (10 ng/ml, BD554603), IL-12 (10 ng/ml, BD554613); (3) Tc17: anti-IL4 (7 µg/ml), anti-IFNγ (7 µg/ml, BD554698), TGFβ1 (10 ng/ml, eBioscience, 14-8348-62), IL-1β (10 ng/ml, BD554602), and IL-6 (50 ng/ml, BD550071). Cultures were activated with 0.5 µg/ml each of fixed anti-CD3 (eBioscience, 16-0037-85) and anti-CD28 (eBioscience, 16-0289-85) and incubated for 7 days at 37°C (Similar protocol was followed for experiments involving naïve CD4+ T-cell differentiation to Th0, Th1. and Th17 conditions). At day 7, cells were washed twice with PBS for suppression assay cultures. An aliquot of cells was washed, re-stimulated and supernatants were aliquoted 48 h later for ELISA assays. In some experiments, an aliquot of cultured cells was used for surface markers and intracellular cytokine staining to assess their state of differentiation.

### ELISA

ELISA was performed on supernatants per manufacturer protocol (eBioscience Human Platinum ELISA Kit for IL-17A (BMS2017). ELISA data were acquired on a BioTek Synergy H1 Hybrid Reader. Gen5 v2.09 was used for software analysis.

### Intracellular Flow Cytometric Cytokine Assays

For surface and intracellular staining on day 7 of *in vitro* differentiation, cells were washed and cultured in media with 2 μL/ml of leukocyte activation cocktail with Golgi Plug (BD, 550583) for 5 h, followed by washing with 0.1% (w/v) sodium azide/phosphate-buffered saline and surface staining with anti-CD3 APC (BioLegend, 300458) and anti-CD8 BV786 (BD, 563823). In some experiments, anti-CD107a PE-Cy7 (BioLegend, 328617) was added during stimulation with cell activation cocktail (BioLegend, 423301) and Monensin (BD Golgi Stop, 554724) (38). Cells were fixed overnight at 4°C followed by permeabilization using fixation/permeabilization kit (eBioscience, 00-5523-00). Intracellular staining was performed using anti-IFNγ AlexaFluor700 (BD, 557995), anti-IL17A PE (eBioscience, 12-7179-42), and anti-Granzyme B APC (Miltenyi, 130-120-703). All cells were resuspended in staining buffer [0.1% (w/v) sodium azide/phosphate-buffered saline] for FACS analysis. Flow cytometric data were acquired on a 4-Laser, 17-color LSRII using BD FACSDiva Software v6.1.3 (Firmware v1.9). FlowJo version 9.1 was used for analysis.

### Flow Cytometric Suppression Assays

CD8+ T-cells from the 7-day differentiation were placed in flow cytometric suppression assays, as described previously (29, 30). Briefly, responder *ex vivo* CD4+ CD25- T-cells were sorted or responder differentiated Th cells were obtained from 7-day cultures and stained with CFSE, followed by culture with irradiated APC and 1 µg/ml of soluble anti-CD3 (eBioscience, 16-0037-85) in the presence or absence of cultured CD8+ Tc cells. On day 7 of suppression culture, cells were stained for anti-CD3 AlexaFlour700 (BD, 557943), anti-CD8 BV786 (BD, 563823), anti-CD4 APC (BD, 555349), and anti-CD25 Pacific Blue (BioLegend, 356130) and flow cytometrically assessed for CD4+ proliferating fraction (CFSE dilution). % proliferation and % suppression were calculated as described previously (29).

### Xenogeneic Graft-Versus-Host Disease (xGVHD)

Xenogeneic graft-vs-host-disease (xGVHD) was induced in female NSG mice (NOD-*scid* *IL2RG* ^null^, strain no. 005557, Jackson Laboratory), as described previously (39, 40). Briefly, *ex vivo*-purified human naïve CD8+ T-cells were first cultured for 7 days in Tc0 and Tc1 differentiation cultures, as described above. On day 7, Tc cells were washed, resuspended with PBS, and injected intravenously into 2 Gy irradiated 6–8-week- old female NSG mice admixed with *ex vivo*-sorted allogeneic bulk CD4+ CD25- T-cells at a 1:1 ratio (10 × 10^6^ CD4 and 10 × 10^6^ CD8 cells/mouse) in the indicated groups. 10 × 10^6^ CD4+ CD25- T-cells/mouse without CD8+ T-cells (1:0 ratio) served as the control group. Some mice also received *ex vivo*-purified bulk CD8+ T-cells (Miltenyi Biotech, 130-045-201) or *in vitro*-differentiated Tc0 or Tc1 cells alone (10 × 10^6^ cells/mouse, 0:1 ratio), representing CD8+ T-cell only groups. Mice were monitored for weight loss up to 15 days post injection. Number of mice per experimental group per experimental replicate is indicated in the figure legend.

### RNA Sequencing/Transcriptome Analysis

Aliquot of cultured CD8+ Tc0 and Tc1A-E cells was flash frozen on day 7, and samples were submitted to the University of Chicago Genomics facility for RNA processing and sequencing. Single-end 50 bp sequencing was performed on the Illumina HiSeq 2000. Pseudoalignment was performed using kallisto (41) with the GRCh38 human genome build and processed using sleuth (v0.30) R package (41). Differential gene expression analysis was performed in the sleuth R package with the Wald test. Unless noted otherwise, differential genes were defined as log2-fold change >1 or <-1 and false discovery rate < 0.05. The significant genes were used for Ingenuity Pathway Analysis (Qiagen) using the same cut-points for significance as inputs.

### Statistics

Graphpad Prism v7.03 (La Jolla, CA) was used for statistical analyses (tests indicated in figure legends). *P* < 0.05 was considered significant. Data represent mean +/- SEM.

### Study Approval

All experiments were performed on PBMC obtained from de-identified leukocyte reduction system (LRS) cones from healthy platelet donors at the University of Iowa DeGowin Blood Center, as approved by the University of Iowa IRB. All mice were kept at the University of Iowa Animal Care Facility under 12-h light/dark cycle, fed ad libitum, humanely cared for, and studied as approved by the University of Iowa’s Institutional Animal Care and Use Committee and in accordance with the National Institutes of Health guide for the care and use of Laboratory animals (NIH Publications no. 8023, revised 1978).

## Results

### Naïve-derived Tc1 Cells Are Deficient in Suppression of CD4+ T-Cell Immune Responses

We have shown that CD8+ T-cells isolated during acute relapse of MS are deficient in their ability to suppress myelin-specific CD4+ T-cell responses (29). Interestingly, the suppressive ability of patient CD8+ T-cells is regained during MS remission (30). We hypothesized that distinct categories of CD8+ cytotoxic T-cell (Tc) lineages may have variable suppressive function against effector CD4+ T-cell responses based on the cytokine milieu under which they were differentiated. To test this hypothesis, we obtained purified human naïve CD8+ T-cells from healthy donor peripheral blood mononuclear cells (PBMC) and differentiated them *in vitro* under the influence of published cytokine combinations (31, 35–37, 42–47) toward Tc0 (control), Tc1 and Tc17 lineages. At day 7 of culture, we confirmed *via* flow cytometry and cytokine ELISA that the differentiated populations of cells exhibited the expected functional phenotypes in terms of cytokine production (**Supplementary Figure 1**). In particular, we confirmed that the Tc0 control condition and the Tc1 condition showed predominantly IFNγ production but almost undetectable IL-17A, whereas the Tc17 conditions exhibited robust IL-17A production. As expected, Tc1 cells showed significantly higher proportion of cells with IFNγ expression (**Supplementary Figure 1**). Similar to previous studies from us and others involving human naïve CD4+ T-cell differentiation (48–50), a small proportion of the differentiated cells showed the presence of signature cytokine at the end of this short-term culture. However, after differentiation in these conditions, other cells within the culture are also known to show functional effects of this differentiation, regardless of production of the specific cytokine (such as IFNγ or IL-17A). Therefore, our studies included the entire population of “Tc1” or “Tc17”-differentiated cells for functional assessment of their suppressive ability, rather than focus on those producing specific cytokines. Thus, the entire Tc0 culture served as an appropriate control for the Tc1 and Tc17 cultures.

**Figure 1:**
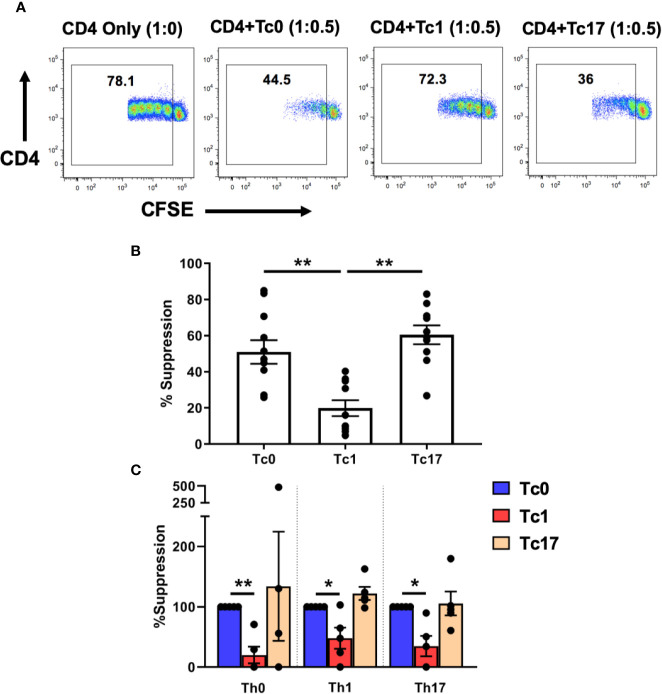
Tc lineages differ in their functional ability to suppress CD4+ immune responses. Cells obtained from Tc0, Tc1, and Tc17 cultures were assessed for suppressor ability against autologous CFSE-stained, *ex vivo*-sorted bulk CD4+ CD25- T-cells **(A**, **B)**. Panel **(A)** shows representative flow cytometric plots demonstrating proliferation of CD4+ T-cells in the presence or absence of indicated Tc cells. Numbers indicate the proportion of cells within the proliferating fraction on day 7 of suppression assay. % suppression was calculated from the proliferation data. Panel **(B)** shows cumulative suppression data, indicating significantly decreased suppression by Tc1 cells (n = 10 independent samples). In Panel **(C)** *ex vivo*-sorted naïve CD4+ T-cells were first differentiated along Th0, Th1, and Th17 lineages for 7 days and then stained with CFSE and subjected to suppression assays using autologous differentiated Tc0, Tc1 and Tc17 cells (n = 5 independent samples). For all three Th types, suppression was normalized to the Tc0 control group and in every case, Tc1 suppression was significantly reduced compared to the Tc0 control. *p < 0.05 and **p < 0.01; paired t test.

We therefore tested the ability of these differentiated Tc cells to suppress CD4+ T-cells using *in vitro* flow cytometric suppression assays, as described previously (29, 30). For these assays, *ex vivo* bulk CD4+ T-cells were stained with CFSE and served as responder T-cells. The cells were stimulated with autologous antigen-presenting cells (APC) and αCD3 and cultured in the presence (or absence) of differentiated autologous CD8+ T-cells. On day 7 of these suppression cultures, the proliferation of CD4+ T-cells was quantified (based on CFSE dilution) and then normalized to the 1:0 “no suppression” control condition (no CD8+ T-cells). As shown in **Figures 1A, B**, we observed that cells from Tc0 and Tc17 cultures had comparable suppressive ability. In contrast, cells from Tc1 cultures showed significantly less suppressive ability against bulk CD4+ T-cells.

We further tested this hypothesis by first generating various Th lineages from CD4+ T-cells and using these as responder cells in suppression assays. Thus, *ex vivo*-purified naïve CD4+ T-cells were cultured for 7 days in the presence of conditions similar to those used for CD8+ T-cells to obtain Th0, Th1, and Th17 cells. These cells were then stained with CFSE and subjected to suppression by autologous naïve-derived Tc0, Tc1, or Tc17 cultures. Similar to our findings with bulk *ex vivo* CD4+ T-cells, we found that cells from Tc0 and Tc17 cultures showed similar suppressive ability against all three Th lineages (**Figure 1C**). Again, Tc1-differentiated cells showed deficient suppressive ability against these Th cells. Therefore, based on these *in vitro* findings, we focused the rest of our studies on understanding the mechanisms responsible for reduced suppressive ability of cells from Tc1 cultures against bulk *ex vivo* CD4+ T-cells.

### Cells From Tc1 Differentiation Conditions Are Suboptimal at Suppressing CD4+ T-Cell-Induced Xenogeneic GVHD *In Vivo*

In order to validate our findings from the *in vitro* suppression assays, we utilized an acute xGVHD model using irradiated NOD-SCID-Gamma (NSG) immunodeficient mice, as described previously (40, 51). Thus, *ex vivo*-purified bulk human CD4+ T-cells were transferred into irradiated NSG mice, either with or without CD8+ T-cells differentiated in Tc0 or Tc1 conditions. Mice were then monitored for induction of acute xGVHD using weight loss as the primary parameter (40, 51–54).

**Figure 2:**
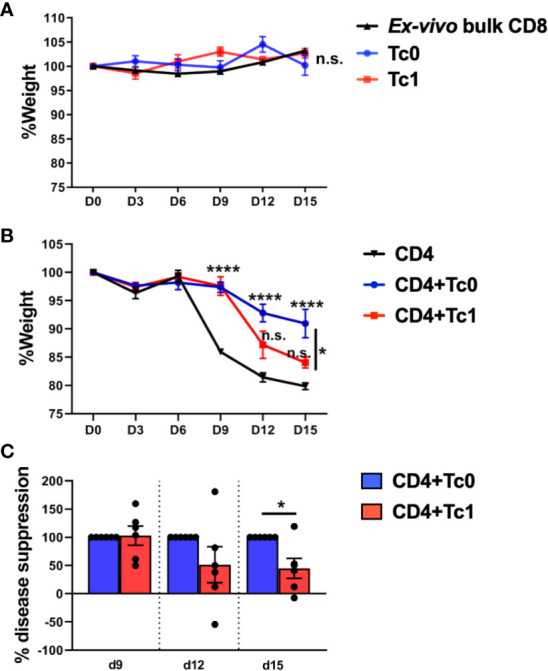
Naïve-derived Tc1 cells have deficient ability to suppress xGVHD *in vivo*. xGVHD was induced in 6–8-week-old female NSG mice by injecting bulk *ex vivo*-purified CD4+CD25- human T-cells with or without *in vitro*-generated, naïve-derived Tc0 (control), and Tc1 cells at a ratio of 1:1 (10 million cells each). Mice were monitored for weight loss over 15 days. Panels **(A, B)** show % weight (normalized to day 0) ± SEM. Panel **(A)** represents absence of xGVHD when only CD8+ T-cells were injected, either as *ex vivo*-purified bulk CD8+ T-cells (two independent replicates; n = 5 mice) or as *in vitro*-differentiated Tc0 or Tc1 cells (1 experiment, n = 3 mice each). Panel **(B)** demonstrates CD4-induced xGVHD with CD4+ T-cells alone or admixed with indicated Tc cells (two independent replicates; n = 6 mice per group). Panel **(C)** shows the same data represented as calculated % disease suppression on d9, d12, and d15 normalized to the Tc0 control group designated as “100% suppression”. *p < 0.05 and ****p < 0.0001; n.s., not significant. **(A, B)** Two-way ANOVA using Tukey’s multiple comparisons test and **(C)** unpaired t-test.

We observed that transfer of *ex vivo*-purified bulk CD8+ T-cells or naïve-derived, *in vitro*-differentiated Tc0 or Tc1 cells did not result in disease (**Figure 2A**). In contrast to bulk or differentiated CD8+ T-cells, bulk CD4+ T-cells resulted in robust disease, as expected (**Figure 2B**). Of note, similar to our findings from *in vitro* suppression assays, the addition of cells from Tc0 control conditions resulted in robust suppression of xGVHD (**Figure 2B**). Interestingly, cells from Tc1 cultures were significantly deficient in their capacity to suppress CD4+ T-cell-driven disease (**Figure 2B**), matching with their deficit in *in vitro* suppression (**Figure 1**). We quantified % disease suppression on days 9, 12, and 15 by comparing % change in weight for the CD4+ T-cell only control group against CD4+Tc0 and CD4+Tc1 groups, normalizing to the Tc0 suppression as 100%. There was strong disease suppression in both the groups by day 9. However, the CD4+Tc1 group progressively lost disease suppressive capability (**Figure 2C**). The experiments were terminated by days 15–18 due to significant weight loss in the control group. These results showed that while cells from the Tc0 control cultures efficiently suppressed CD4+ T-cell driven pathology *in vivo*, cells from Tc1 cultures had an intrinsic suppressive defect.

### Exposure to IL-12 During CD8+ T-Cell Differentiation Results in Suboptimal Suppressive Ability With Dysregulated Induction of Cytotoxicity-Related Molecules

**Figure 3:**
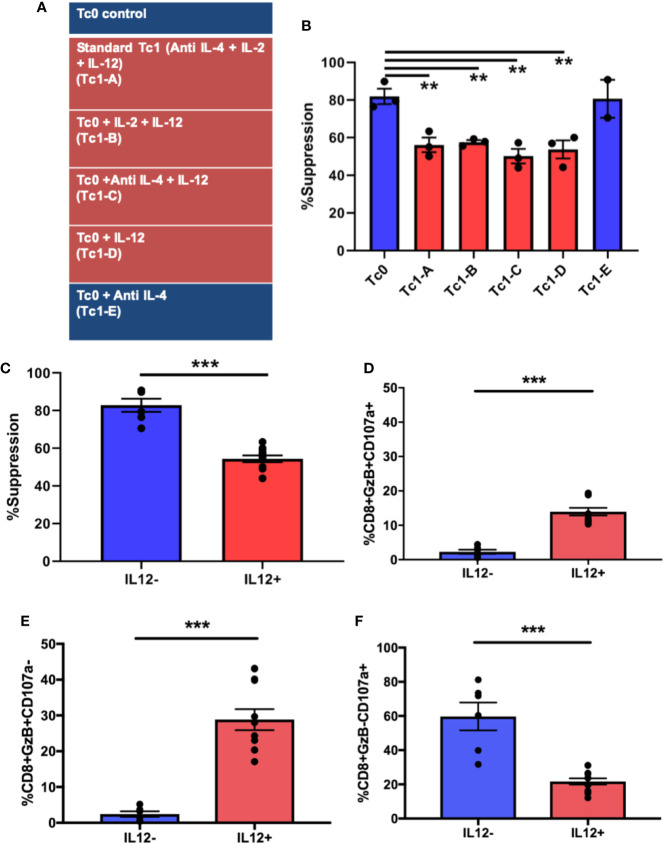
Exposure to IL-12 induces CD8+ T-cells characterized by suboptimal suppressive ability with dysregulated induction of cytotoxicity-related molecules. Six different culture conditions **(A)** were used to differentiate *ex vivo*-purified naïve CD8+ T-cells. On day 7, these Tc cells were assessed for suppressor ability against autologous CD4+CD25- T-cells. Panel **(B)** demonstrates %suppression for each of the conditions. Panel **(C)** shows the same data combined into two groups: cultures containing IL-12 (n = 12) and those without (n = 6). In parallel, cells from the 7-day cultures were stimulated with BioLegend Cell Activation cocktail with Monensin and anti-CD107a for 5h followed by intracellular staining for granzyme B. %GzB+CD107a+ dual positive CD8+ T-cells were quantified within the indicated groups **(D)**, as were GzB+ **(E)** and CD107a+ **(F)** single-positive cells. **p < 0.01 and ***p < 0.001. **(B)** One-way ANOVA and **(C–F)** Mann-Whitney test.

We next asked whether the functional differences in Tc1 CD8+ T-cells can be attributed to specific cytokines that they are exposed to during their differentiation. In the Tc1 differentiation condition, IL-12 was the dominant pro-inflammatory cytokine and seemed potentially accountable for mediating the loss of suppressive ability. We tested this hypothesis by setting up 6 different CD8+ T-cell culture conditions (**Figure 3A**). Thus, in addition to the media control (Tc0) and the standard Tc1 condition (now designated Tc1-A), we added 4 other variations that included different combinations of the same factors (IL-2, IL-12 and anti-IL-4). First, we utilized these differentiated CD8+ T-cells from all 6 conditions in suppression assays against autologous CD4+ T-cells. We observed that the 4 conditions that contained IL-12 (Tc1A-D) resulted in a significant loss of suppressive ability, when compared to the Tc0 condition (**Figure 3B**). When we grouped the conditions as containing or not containing IL-12, this confirmed that the populations exposed to exogenous IL-12 showed significantly lower suppressive ability (**Figure 3C**).

In order to investigate the cytotoxic potential of these cells, they were surface-stained for the degranulation marker lysosomal-associated membrane protein 1 (LAMP1 or CD107a) (38, 55–57), followed by intracellular staining for the cytotoxic molecule, granzyme B (GzB). As shown in **Figure 3D**, the IL-12-exposed group exhibited a significantly greater proportion of dual-positive (granzyme B+CD107a+) cells compared to the group not exposed to IL-12 (including Tc0). In prior work, we have shown that suppressive ability of CD8+ T-cells is linked with their cytotoxic functions (9, 30). Therefore, it was somewhat unexpected that suboptimally suppressive Tc cells would show a greater proportion of cells expressing granzyme B. We therefore evaluated the two properties (degranulation vs. granzyme production) individually by quantifying single-positive and total-positive cells for each marker (representative dot plots shown in **Supplementary Figure 2A**). In this analysis, we saw that cells from IL-12-containing cultures showed significantly greater proportion of cells producing granzyme B, quantified either as single positive (**Figure 3E**) or as total positive (**Supplementary Figure 2B**), but demonstrated a significant reduction in the proportion of cells expressing surface CD107a (**Figure 3F** and **Supplementary Figure 2B**). Additionally, in linear regression analysis, whereas the cells from the IL-12(-) cultures demonstrated the expected positive correlation between suppression and degranulation, such correlation was lost in IL-12(+) cultures (**Supplementary Figure 2C**). Collectively, these findings suggest that exposure of naïve CD8+ T-cells to IL-12 may result in enhanced production of cytotoxic molecules but an inhibition in the granule transport and/or degranulation machinery, potentially translating to a reduced cytotoxic/suppressor capacity.

### Transcriptome Analysis of Naïve-Derived Tc Lineages Reveals Potentially Targetable Pathways to Reverse the IL-12- Induced Suppressive Deficit

In an effort to dissect out the mechanisms responsible for these IL-12-induced functional changes, we differentiated naïve CD8+ T-cells for 7 days in various conditions depicted in **Figure 3A**, followed by RNAseq transcriptome analysis. We then compared differential gene expression between the groups that received or did not receive IL-12 during differentiation. First, we asked whether there was any evidence of greater apoptotic potential among the IL-12-exposed cells that could explain their inability to optimally suppress their targets. As shown in **Supplementary Figure 3**, we did not observe any significant differences in either pro-apoptotic (*BCL2L11*/Bim, *BID*, and *BAD*) or pro-survival factors (*BCL2* and *BCL2L1*/Bcl-xl) (58).

**Figure 4:**
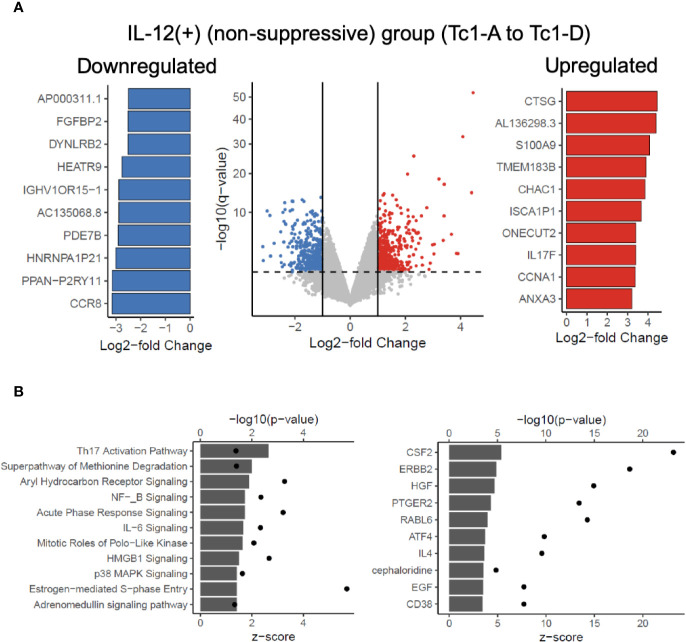
Transcriptome analysis reveals potentially targetable genes and pathways to reverse IL-12-induced suppressive deficit. RNAseq analysis was performed on 7-day differentiated Tc subtypes shown in **Figure 3A**. Panel **(A)** shows significant differential genes in the IL-12(+) (“non-suppressive”) group when compared against IL-12(-) (suppressive) group. Significant defined as log2-fold change > 1 or < -1 and adjusted p-value < 0.05. Top 10 significant differential genes upregulated (red) or downregulated (blue) as defined by log2-fold change are shown. Panel **(B)** depicts the IPA analysis of upregulated canonical pathways and upregulated predicted upstream regulators in the IL-12(+) (non-suppressive) group with z-score as bar charts and points as –log10(p-values).

Next, we asked which genes were differentially regulated in the IL-12(+) group, relative to the IL-12(-) group. **Figure 4A** shows a volcano plot of differentially expressed genes (451 significantly upregulated shown in red and 441 significantly downregulated shown in blue), with the top 10 significantly up- and downregulated genes shown in the bar graphs. In addition to evaluating the two broad groups, IL-12(+) and IL-12(-), we also compared differential expression between each of the IL-12-containing experimental conditions, relative to the Tc0 control (**Supplementary Figure 4**). In this manner, we were better able to tease out the specific effects of IL-12 within our *in vitro* system. Across all four comparisons, we saw a number of genes significantly up- and downregulated. The 31 common genes upregulated and 19 common genes downregulated between individual comparisons are represented in heatmaps in **Supplementary Figure 4**. Importantly, we performed Ingenuity Pathway Analysis (IPA) by utilizing the differential expression comparisons between IL-12-exposed (non-suppressive) group versus the IL-12-unexposed (suppressive) group. We saw 25 canonical pathways significantly upregulated in the IL-12(+) non-suppressive group (**Figure 4B**). IPA also performs estimated enrichments for upstream regulators of distinct genetic programs. We examined the upstream regulators with increased and decreased enrichment (**Figure 4B** and **Supplementary Figure 5**) across the IL-12(+) group. Interestingly, we found the highest increase in genetic expression patterns associated with the cytokine GM-CSF (*Csf2*), among other cytokines and transcription factors. These results suggested that IL-12 could act directly on naïve CD8^+^ T-cells to induce GM-CSF-related pathways.

Finally, in the context of our findings related to the disconnect between the production of granzyme B vs degranulation (**Figure 3**), we directly evaluated whether any of the genes associated with cytotoxicity and degranulation were different between the two groups. We observed significant differences in *IFNG*, *GZMB*, *STX11*, *VAMP7*, *and LYST* genes (**Supplementary Figure 6**). Corroborating our flow cytometry data, we found upregulation of *IFNG* and *GZMB* in the IL-12(+) group. There were no differences in message for CD107a or perforin. Interestingly, the *LYST* gene was significantly downregulated in IL-12-exposed cells (**Supplementary Figure 6**). The function of *LYST* (lysosomal trafficking regulator) is incompletely understood, but its mutation is known to cause Chediak-Higashi syndrome and some forms of hemophagocytic lymphohistiocytosis, characterized by defective lymphocyte degranulation due to changes in the morphology and function of secretory lysosomes (59–62). It is thought to be required for the maturation of cytotoxic granules into exocytosis-competent secretory granules (59), and therefore, the downregulation of this gene may offer a mechanistic explanation for the inhibited degranulation seen in **Figure 3**.

## Discussion

Several studies from our group and others have underscored the importance of CD8+ T-cells as regulators of destructive effector CD4+ T-cell responses during immune-mediated disease (8, 9, 14, 26). In these situations, deficient CD8-mediated regulation may be associated with greater disease severity, such as relapses of multiple sclerosis (29, 30). Therefore, it is critical to understand the genesis of such regulatory deficiency in order to devise intervention strategies.

In the current study, we addressed the hypothesis that cytokine-milieu-driven differentiation of CD8+ T-cells along various lineages results in differential ability to regulate effector CD4+ T-cell responses. While dissecting this biology, we have uncovered several fundamental concepts. First, we show that CD8+ T-cells differentiating under “Tc1 conditions” are significantly inhibited in their ability to suppress CD4+ effector T-cells, compared to either Tc0 controls or cells cultured under Tc17 conditions. Importantly, we show this using not only *in vitro* suppression assays but also in an *in vivo* model of xenogeneic GVHD in humanized NSG mice, validating the disease relevance of this finding. Further, we demonstrate that IL-12 exposure is the main driver of this phenotype and likely influences CD8+ suppressor function through induction of a dysregulated degranulation process affecting cytotoxicity. Finally, we conduct transcriptome analysis of these differentiated Tc cells, revealing the induction of multiple pro-inflammatory pathways, which could be potentially targetable for disease intervention. These hypothesis-generating data can help hone future investigation in the context of multiple clinical settings.

The inflammatory role of IL-12 revealed in the mid-1990s provided the rationale to develop the human IL-12-neutralizing antibodies, such as ustekinumab and briakinumab, which have been evaluated in a number of immune-mediated diseases like psoriasis, rheumatoid arthritis and multiple sclerosis, with psoriasis seeing the most advanced clinical development (63). High levels of IL-12 are detected in the aqueous humor, serum and synovial fluid of patients with different autoimmune conditions like autoimmune uveitis, multiple sclerosis and rheumatoid arthritis and are correlated with disease activity (64–67). This underscores the involvement of IL-12 in modulating immune-cell subsets in autoimmune diseases. Our findings suggest that IL-12 may exert its “pro-inflammatory” role, partly through inhibition of immune suppressive mechanisms. In particular, we observed that IL-12-induced Tc1 cells were suboptimal inhibitors of pathogenic CD4+ T-cells. This also speaks to a fundamental aspect of Tc1 differentiation wherein acquisition of certain effector functions is associated with inhibition of regulatory function.

In the current study, our *in vitro* differentiation cultures contained purified CD8+ T-cells, devoid of other immune cell types, such as APC or other lymphocytes. Therefore, our observations provide insights into a CD8+ T-cell-intrinsic processes downstream of IL-12 signaling in the context of global anti-CD3/anti-CD28-mediated stimulation. Some of our prior studies that have utilized PBMC cultures and specific autoantigens for the generation of autoregulatory CD8+ T-cells have shown the ability for IL-12 to enhance the ability of these cells to modulate autoantigen-specific effector responses (30). These differences suggest pleiotropic effects of this cytokine based on the overall immune environment, strength of antigenic stimulus and potentially the dose of cytokines in the inflammatory microenvironment. It will be important to dissect these features in future studies.

In a recent report, we have shown that naïve CD4+ T-cells attain distinct levels of resistance to CD8-mediated suppression (50). There we observed that “Th1” conditions rendered CD4+ T-cells more sensitive to suppression, whereas Th17 conditions resulted a cells that were significantly resistant to suppression. It is interesting that distinct cytokine milieus are responsible for inducing CD4 resistance (Th17 conditions) vs. CD8 suppressive deficit (Tc1 conditions). It will be critical to understand how various immune cell types respond within co-cultures that mimic a common set of *in vivo* inflammatory signals.

In the current study, we performed RNAseq and transcriptome analysis to gain insights into the mechanistic changes that may influence immune-suppressive behavior. First, we confirmed that pro-apoptotic (*BCL2L11*/Bim, *BID* and *BAD*) and pro-survival (*BCL2* and *BCL2L1*/Bcl-xl) factors (58) were not altered in IL-12-induced Tc1 cultures (**Supplementary Figure 3**). Next, we looked at IL-12-mediated effects on maintaining cellular proliferation and activation. Prior studies have shown IL-12 to promote the expression of cell cycle associated transcripts, enhancing division of activated CD8+ T-cells by maintaining IL-2 signaling *in vivo* (58). IPA analysis of our *in vitro* data revealed cell-cycle related pathways like ‘estrogen mediated S phase entry’ significantly upregulated in IL-12(+) culture group (**Figure 4B**), and we also found Tc1 cultures to express the greatest proportion of CD25+ cells at the end of suppression assays (data not shown). We looked at modulation of markers associated with terminal differentiation and exhaustion and observed significant downregulation of *CD160* and *CTLA-4* whereas significant upregulation of *LAG3* in IL-12-induced Tc1 cultures (**Supplementary Figure 7**).

In addition, we observed upregulation of several pro-inflammatory pathways in Tc1 cells, with an interesting involvement of GM-CSF and its associated pathways – NF-κB, MAPK, HMGB1 in the IL12-exposed non-suppressive groups. The role of CD4+ T-cell-derived GM-CSF has been implicated in autoimmune tissue inflammation in mouse models of neuroinflammation, arthritis, and myocarditis (68–71). Murine Th17 cells were identified as the chief source of GM-CSF, however multiple mouse and human studies have shown IL-12 to modulate Th1 cells and produce GM-CSF *via* an IL-23-independent pathway (72, 73). GM-CSF+ CD8+ T-cells have been shown to predominate in MS lesions (74). However, the role of CD8+ T-cell-derived GM-CSF and its regulation remains poorly understood. Our studies indicate activity of GM-CSF-related pathways in IL-12-differentiated human CD8+ T-cells, which will be an interesting aspect of future investigation, including whether this has a bearing on their disease regulatory role.

Finally, we evaluated the status of cytotoxic pathways in IL-12-induced Tc1 cells. We have shown in prior studies that CD8-mediated suppressor function relies on granzyme- and perforin-mediated cytotoxic killing of pathogenic CD4+ T-cells or pro-inflammatory APCs in autoimmune disease, both in mice and humans (9, 26, 29, 30). Therefore, we quantified granzyme B production as well as degranulation (as measured by surface CD107a) in Tc1 cells. While we observed higher proportion of granzyme B/CD107a double-positive cells within IL-12 exposed Tc1 populations, upon looking at these two properties individually, we saw that IL-12(+) cultures produced robust granzyme B, but demonstrated a significant reduction in surface CD107a expression (**Figures 3E, F** and **Supplementary Figure 2B**). Additionally, in a linear regression plot, the IL-12(-) cultures demonstrated a positive correlation between suppression and degranulation. Interestingly, this correlation was lost in IL-12(+) cultures with notable phenotypic clustering (**Supplementary Figure 2C**). Thus, it seems plausible that exogenous IL-12 within the microenvironment may render dysfunction in the granule transport and/or degranulation machinery leading to cytotoxic molecules being held up within the CD8+ Tc1-cell. In contrast, Tc0 cells may efficiently pump cytotoxic content outside the cells upon activation. We attempted to corroborate this functional observation within our transcriptome data. Of the various cytotoxicity-related genes (including perforin and CD107a), we found significant differences in *IFNG*, *GZMB*, *STX11*, *VAMP7*, *and LYST* genes. Importantly, granzyme B message was significantly higher in the IL-12-exposed group, with significant downregulation of *LYST* gene, which functions as a lysosomal trafficking regulator in lymphocytes (59). This discordant behavior in CD8+ T-cells has been shown in disorders associated with mutations in *LYST* gene like Chediak-Higashi syndrome and some forms of hemophagocytic lymphohistiocytosis (62, 75), characterized by defects in proteins regulating cytotoxic lymphocyte degranulation and shown to reduce surface CD107a expression (76–78). Surface CD107a has also been shown to protect NK cells from degranulation-associated damage (79). Overall, our studies indicate a novel angle of IL-12 acting directly on naïve CD8+ T-cells to induce a phenotype characterized by deficient immune-suppressive behavior, which may be the result of dysregulated cytotoxicity/granule transport. Prior studies have shown that this defect could be rescued by expression of effectors of lytic granule exocytosis, like Munc-13-4, Rab27a and Slp3 in cytotoxic T-lymphocytes (59). This may lead the way for potential translation of these types of interventions as a therapeutic strategy for autoimmune diseases.

To summarize, we have shown the novel finding that IL-12-mediated Tc1 differentiation results in reduced immune suppressive ability in CD8+ T-cells and have demonstrated plausible mechanistic explanations which will provide the basis for future investigation.

## Data Availability Statement

The datasets presented in this study can be found in online repositories. The names of the repository/repositories and accession number(s) can be found at: https://www.ncbi.nlm.nih.gov/geo/, GSE151204.

## Ethics Statement

The animal study was reviewed and approved by University of Iowa IACUC.

## Author Contributions

PR, SS, and NK contributed to conception and design of the study. PR, AB, NB, MC, SS, and SS-T performed the experiments and acquired and analyzed data. PR and NK organized the datasets and wrote the first draft of the manuscript. All authors contributed to manuscript revision, read, and approved the submitted version.

## Funding

This work was supported, in part, by grant awards from the NIH to NJK (R01 AI121567) and NB (F30 CA29655).

## Conflict of Interest

The authors declare that the research was conducted in the absence of any commercial or financial relationships that could be construed as a potential conflict of interest.

## Acknowledgments

The authors would like to thank Drs. Alexander Boyden, Ashutosh Mangalam, Scott Lieberman, Ali Jabbari, and Chakrapani Vemulawada for the helpful discussions and advice.
