# Supplementary material for: IL-12-Induced Immune Suppressive Deficit During CD8+ T-Cell Differentiation
Source: Front Immunol. 2020 Oct 28;11:568630. doi: 10.3389/fimmu.2020.568630 (PMC7657266; doi:10.3389/fimmu.2020.568630)
Supplement: Supplementary file 1 [file DataSheet_1.pdf]

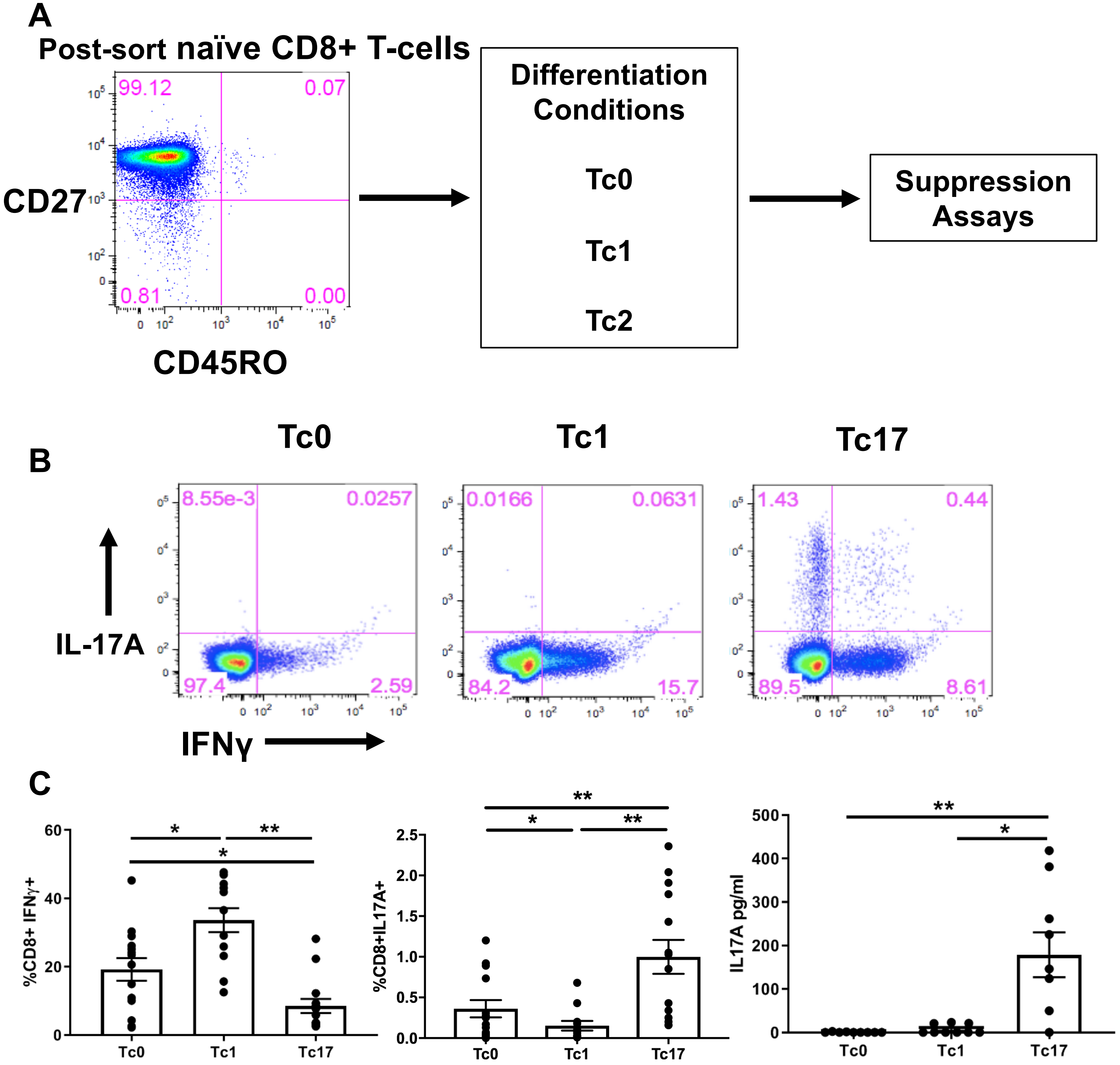

Supplementary Figure 1: Cytokine driven in-vitro differentiation of human naïve CD8+ T-cells. Panel A depicts the overall design. Healthy human naïve CD8+ T-cells were sorted from PBMC using the naïve CD8+ T-cell isolation kit. Flow cytometry dotplot shows representative sorted cells (>99% naïve). These cells were activated for 7 days with anti-CD3+anti-CD28 in the presence of Tc0 (media control), Tc1 and Tc17 differentiation conditions and were then used as suppressors in flow cytometric suppression assays, using autologous CD4+ effectors and irradiated APC. Panels B and C demonstrate cytokine expression by the indicated Tc cells post-7-day differentiation cultures. Panel B shows representative cytokine flow cytometry staining of 5h re-stimulated cells. Panel C shows cumulative cytokine flow cytometry data for IFNγ and IL17A (left two panels) and cumulative data from IL-17A ELISA assays on supernatants post 48h re-stimulation after day 7 (right panel).

*=$p<0.05$, **$p<0.01$; paired t-tests

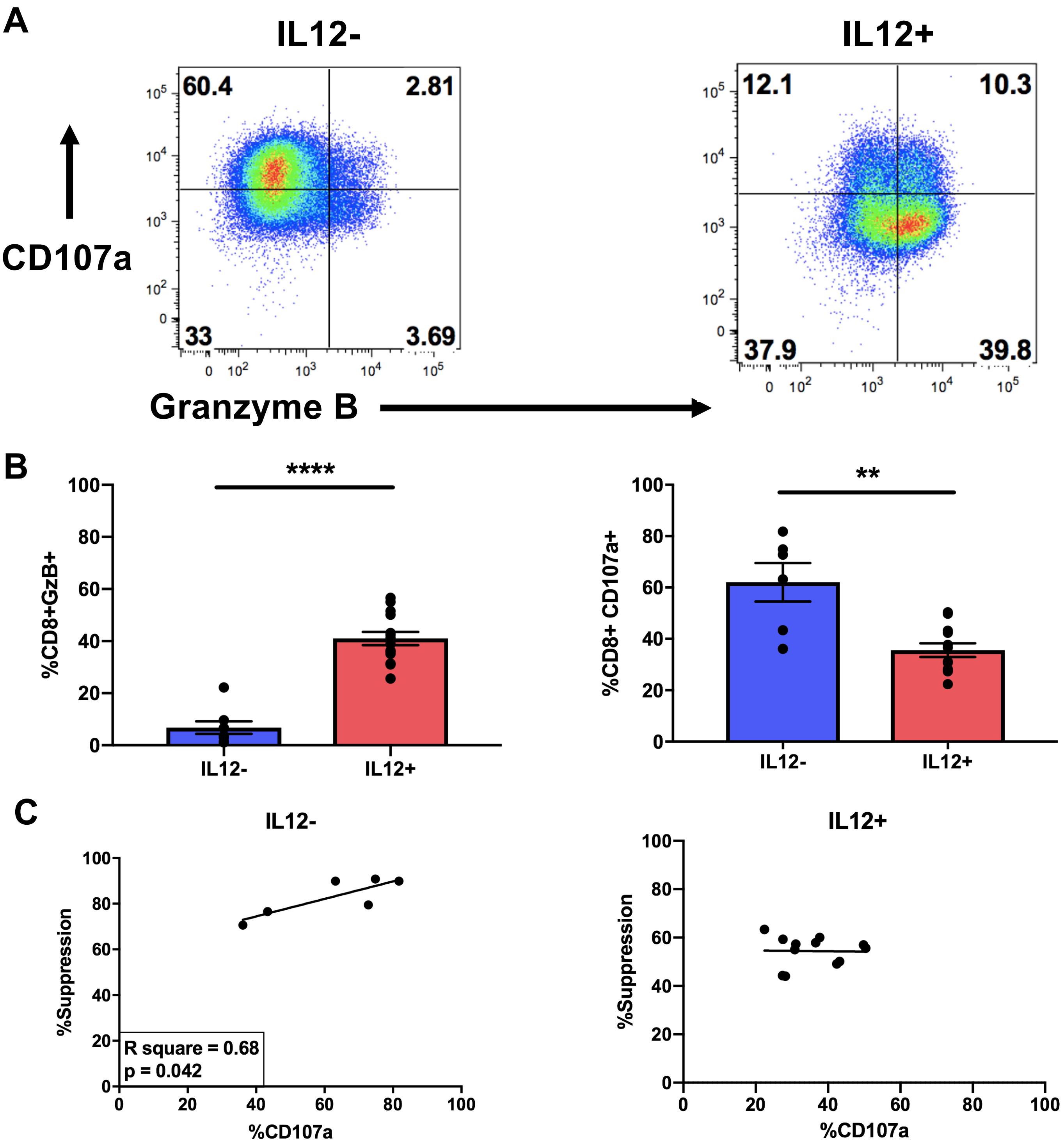

Supplementary Figure 2: Naïve CD8+ T-cell exposure to IL-12 induces higher production of intracellular granzyme and significant reduction in surface CD107a expression. Tc lineage differentiation conditions were altered in six different ways with or without IL-12 (as shown in Fig. 3A). On day 7, cells were stimulated with BioLegend Cell Activation cocktail with Monensin and anti-CD107a for 5h followed by intracellular staining for granzyme b (GzB). Panel A. Representative flow cytometric plots demonstrating granzyme B vs CD107a on Tc-cells in the absence or presence of IL-12. Panel B. Cumulative data from multiple donor samples for total GzB or CD107a expression. **p<0.01, ****p<0.0001; Mann-Whitney test. Panel C. Linear regression plots of IL12(-) and IL12(+) groups showing degranulation marker %CD107a versus %Suppression.

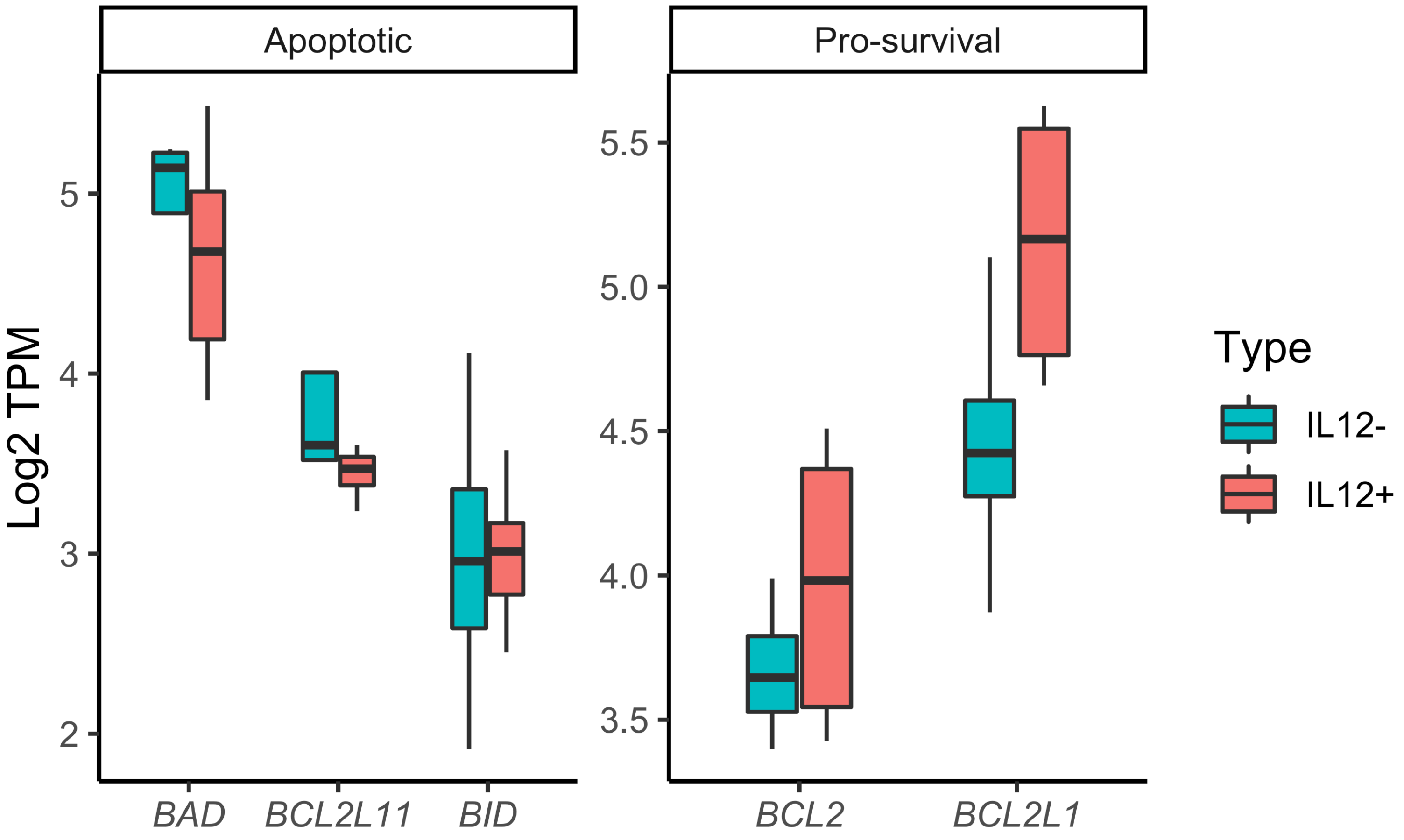

Supplementary Figure 3: Apoptotic and pro-survival genes are not significantly different between CD8+ T-cells differentiated in the presence or absence of IL-12. RNASeq analysis was performed on 7-day differentiated Tc subtypes shown in Fig. 3A. Figure shows Log2(TPM+1) mRNA level of apoptotic (*BAD*, B*CL2L11*, and *BID*) and pro-survival (*BCL2* and *BCL2L1*) genes between IL12(-) and IL12(+) groups.

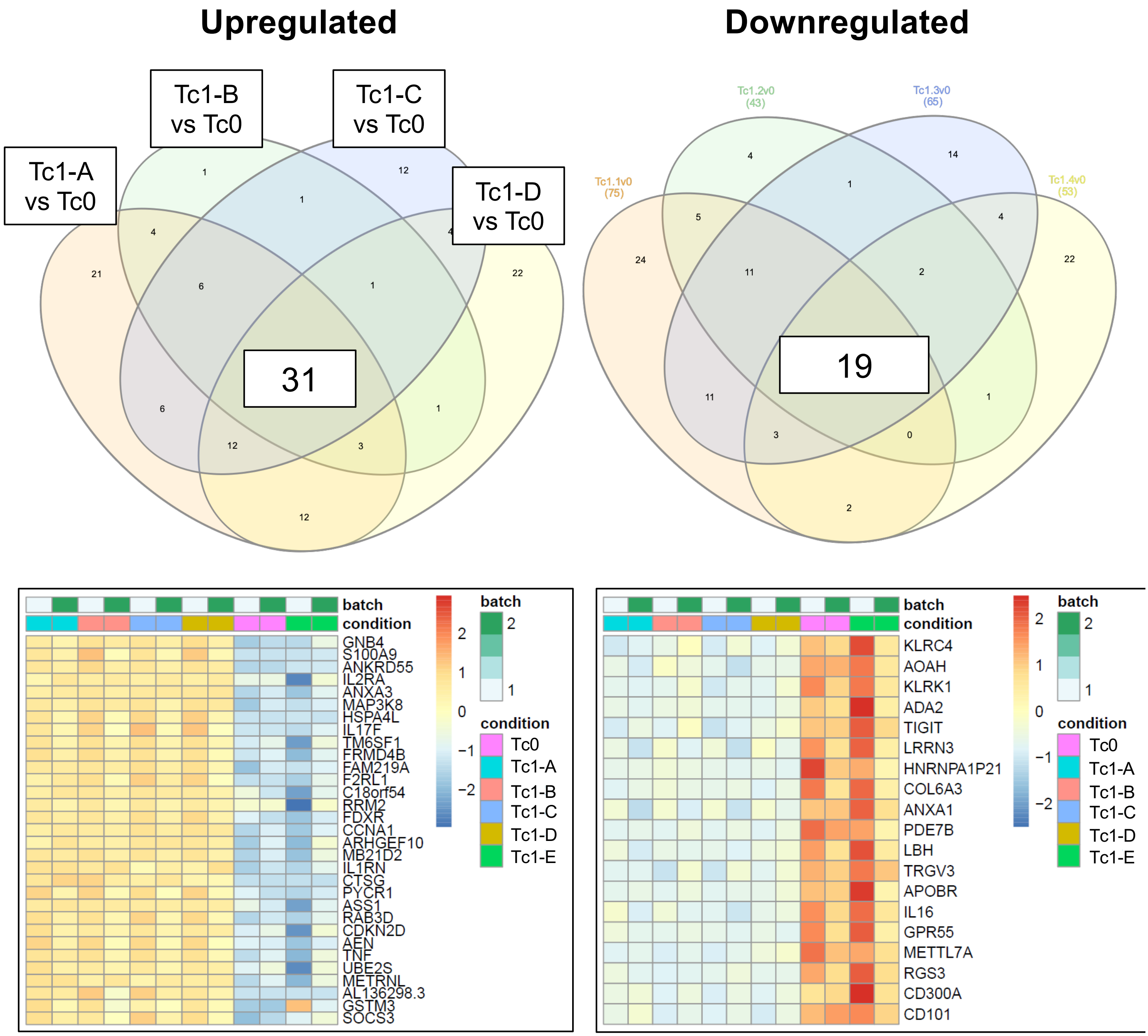

Supplementary Figure 4: Transcriptome analysis reveals common up- and down-regulated genes in various IL-12-induced Tc lineages associated with suppressive deficit. RNASeq was performed on 7-day differentiated Tc subtypes (shown in Fig. 3A). Figure shows Venn diagrams of the upregulated and downregulated genes across individual IL-12(+) (non-suppressive) conditions compared with IL-12(-) Tc0 control (suppressive) and accompanying heatmap of the 31 and 19 genes common between the conditions.

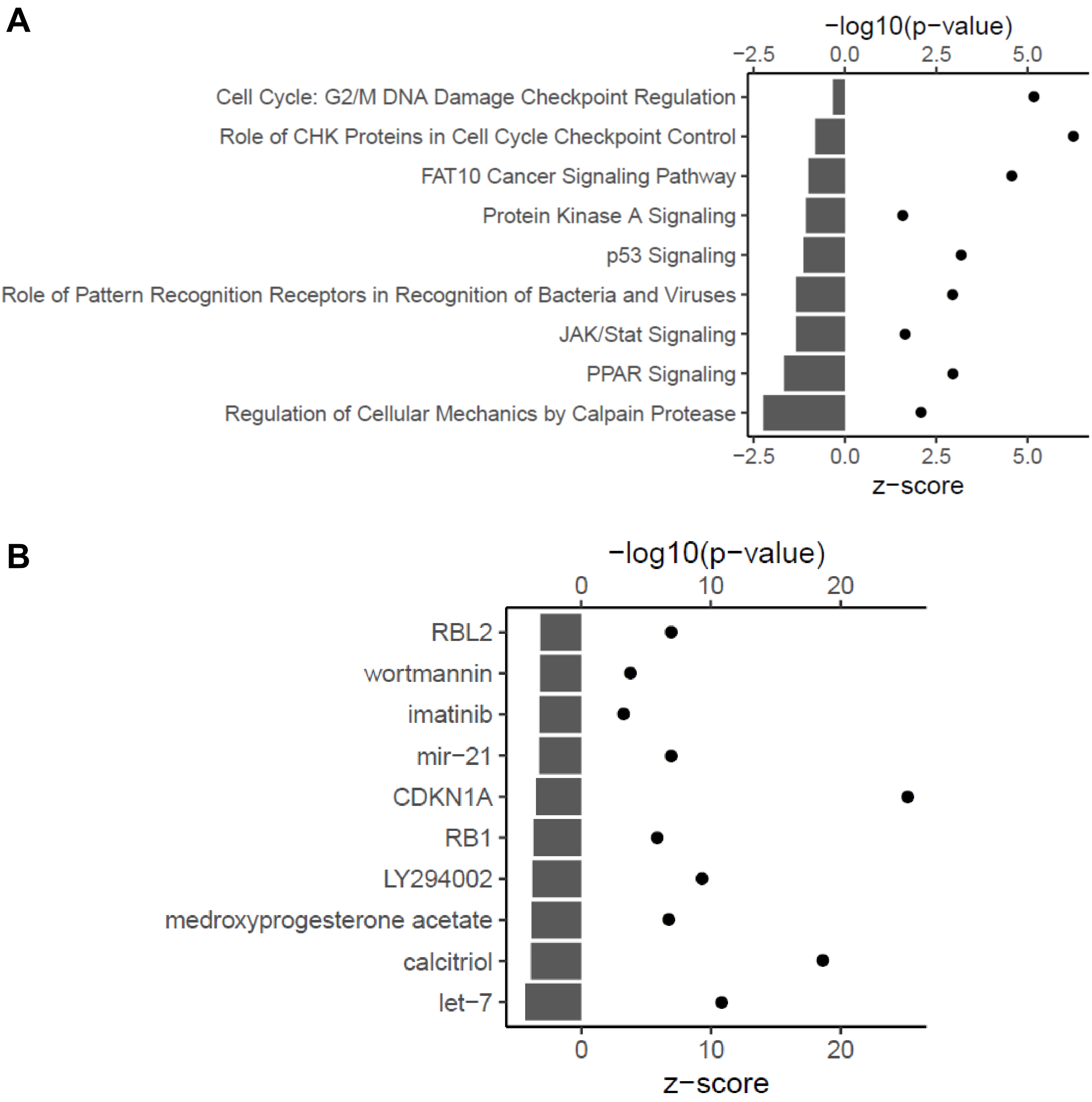

Supplementary Figure 5: Transcriptome analysis reveals potentially targetable pathways to reverse IL-12-induced suppressive deficit. RNAseq was performed on 7-day differentiated Tc subtypes shown in Fig. 3A. Graphs show IPA analysis of downregulated canonical pathways with z-score as bar charts and points as –log10(p-values) (Panel A) and downregulated predicted upstream regulators (Panel B) in the IL-12(+) non-suppressive group.

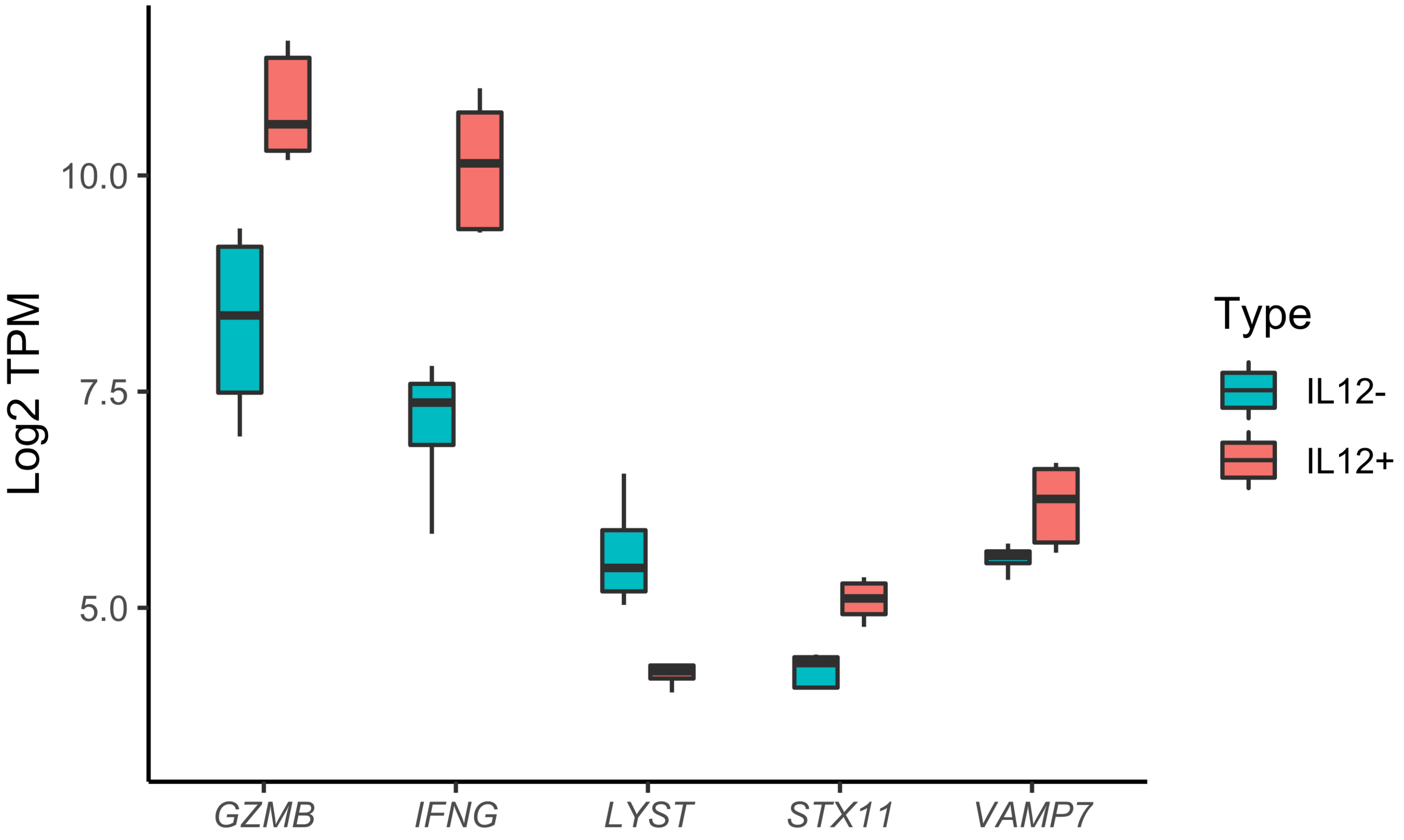

Supplementary Figure 6: Genes associated with cytotoxic/degranulatory machinery are significantly different between CD8+ T-cells differentiated in the presence or absence of IL-12. RNASeq analysis was performed on 7-day differentiated Tc subtypes shown in Fig. 3A. Figure shows Log2(TPM+1) mRNA level of significant cytotoxic and degranulatory genes between IL-12(-) and IL-12(+) groups. Adjusted p (q) values are: *IFNG*: 5.89e-10, *GZMB*: 6.67e-6, *STX11*: 5.68e-5, *VAMP7*: 4.36e-2, *LYST*: 2.60e-7.

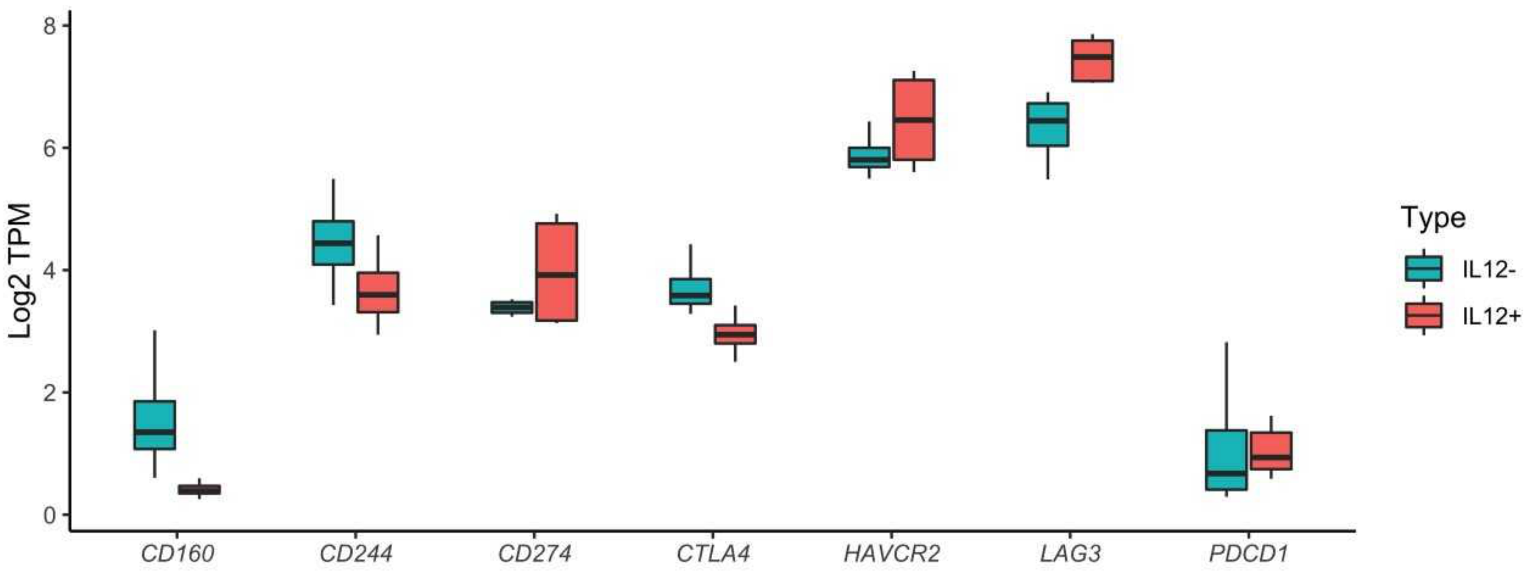

Supplementary Figure 7: Genes associated with terminal differentiation and exhaustion are significantly different between CD8+ T-cells differentiated in the presence or absence of IL-12. RNASeq analysis was performed on 7-day differentiated Tc subtypes shown in Fig. 3A. Figure shows Log2(TPM+1) mRNA level of terminal differentiation and exhaustion genes between IL-12(-) and IL-12(+) groups. Adjusted p (q) values are: *CD160*: 1.66e-3, *CD244*: not significant (n.s.), *CD274* (PD-L1*)*: n.s., *CTLA-4*: 5.0e-2, *HAVCR2* (TIM-3): n.s., *LAG3*: 1.18e-3, *PDCD1* (PD-1): n.s.
